# Resuscitative endovascular balloon occlusion of the aorta: the postpartum haemorrhage perspective

**DOI:** 10.1186/s13054-022-03942-0

**Published:** 2022-03-11

**Authors:** Jostein Rødseth Brede, Edmund Søvik, Marius Rehn

**Affiliations:** 1 Department of Emergency Medicine and Pre-Hospital Services, St. Olav’s University Hospital, Prinsesse Kristinas Gate 3, 7030 Trondheim, Norway; 2 Department of Research and Development, Norwegian Air Ambulance Foundation, Oslo, Norway; 3 Department of Anesthesiology and Intensive Care Medicine, St. Olav’s University Hospital, Trondheim, Norway; 4 Department of Circulation and Medical Imaging, Faculty of Medicine and Health Sciences, Norwegian University of Science and Technology (NTNU), Trondheim, Norway; 5 Department of Radiology and Nuclear Medicine, St. Olav’s University Hospital, Trondheim, Norway; 6 Division of Prehospital Services, Air Ambulance Department, Oslo University Hospital, Oslo, Norway; 7 Faculty of Health Sciences, University of Stavanger, Stavanger, Norway

**Keywords:** REBOA, Postpartum haemorrhage, PPH, Aortic occlusion

## Background

**Fig. 1:**
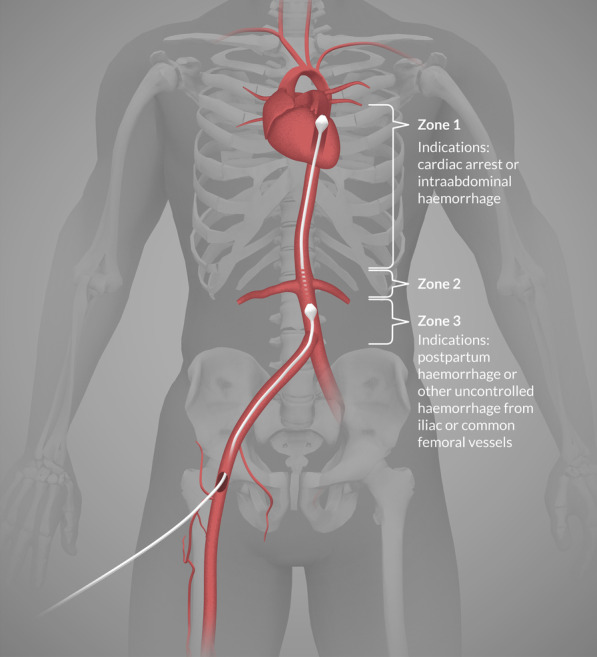
Aortic zones and indications for occlusion

Resuscitative endovascular balloon occlusion of the aorta (REBOA) has become a recognised intervention in management of non-compressible traumatic haemorrhage [1] and ruptured aortic aneurisms [2] (Fig. 1). Additionally, it is used to limit blood loss in cases of post-partum haemorrhage (PPH) [3–5], and is lately considered a possible experimental adjunct in management of non-traumatic cardiac arrest [6] currently investigated in a prehospital randomized controlled trial [7].

Application of REBOA in management of non-compressible traumatic haemorrhage is well-described. However, this aetiology for potential use may be outnumbered. Postpartum haemorrhage (PPH) remain a global health problem, responsible for 8% of maternal deaths. Rates are increasing and in the USA approximately 40/10 000 deliveries suffer from PPH [8, 9].

A recent gap-analysis assessing REBOA eligible patients with major haemorrhage in Norway used blood bank data to identify patients subject to massive transfusions [10]. The aetiology was non-traumatic in 83% of cases, dominated by PPH, followed by ruptured abdominal aortic aneurysm. This carries relevance for REBOA also in regions with low trauma burden.

## Main text

Approximately, 70% of PPH is due to uterine atony, and treatment include uterine massage, B-Lynch suture and intrauterine balloon [9]. However, these interventions may be insufficient, making emergency hysterectomy a life-saving procedure. Globally, the incidence of emergency peripartum hysterectomy varies from 0.2 to 5.1 per 1000 deliveries, with increasing rates [11]. An emergent use of REBOA in management of PPH, including prophylactic strategies in placenta accreta, has been described [3–5]. However, a Cochrane database review on the use of mechanical interventions for treating PPH [12] and a recent review on management of PPH in the New England Journal of Medicine failed to mention REBOA as possible adjunct [9]. REBOA is a highly invasive intervention carrying a potential for serious complications and should not be applied if measures such as intrauterine balloon or uterine massage is sufficient. Nevertheless, in countries with limited access to blood products, REBOA may save lives in PPH where traditional management fails.

In high-income countries, with easy access to blood products, a difference in survival may be difficult to demonstrate. However, we suggest that survival may not represent the only relevant endpoint. REBOA in PPH may reduce hysterectomy rates, a surgical procedure considered catastrophic for any young female and potentially reduce transfusion requirements. A randomised controlled trial on REBOA in PPH with additional endpoints than survival, where hysterectomy rates, transfusion requirements and serious adverse events are investigated, is called for.

Not only in trauma centres, but also in hospitals with obstetric departments, REBOA should be considered an emergency procedure to be immediately available 24/7 by physicians trained in ultrasound-guided and fluoroscopy-free Seldinger technique. Local considerations will decide whether the REBOA is placed by an emergency physician, anaesthesiologist, obstetricians, interventional radiologist or the general surgeon.

## Conclusions

REBOA carries more indications than trauma and should be increasingly considered and evaluated in management of PPH. REBOA may not only save a life, it might also save a uterus.

## Data Availability

Not applicable.

## Abbreviations

REBOA: Resuscitative endovascular balloon occlusion of the aorta
PPH: Postpartum haemorrhage
